# Investigating how the accuracy of teacher expectations of pupil performance relate to socioeconomic and genetic factors

**DOI:** 10.1038/s41598-022-11347-w

**Published:** 2022-05-03

**Authors:** Ciarrah-Jane Shannon Barry, Neil M. Davies, Tim T. Morris

**Affiliations:** 1 Medical Research Council Integrative Epidemiology Unit at the University of Bristol, Bristol, BS8 2BN UK; 2 Population Health Sciences, Bristol Medical School, University of Bristol, Barley House, Oakfield Grove, Bristol, BS8 2BN UK; 3 K.G. Jebsen Center for Genetic Epidemiology, Department of Public Health and Nursing, NTNU, Norwegian University of Science and Technology, Trondheim, Norway

**Keywords:** Genetics, Cognitive neuroscience, Risk factors

## Abstract

Teacher expectations of pupil ability can influence educational progression, impacting subsequent streaming and exam level. Systematic discrepancies between teacher expectations of pupil achievement may therefore have a detrimental effect on children’s education. Associations between socioeconomic and demographic factors with teacher expectation accuracy have been demonstrated, but it is not known how teacher expectations of achievement may relate to genetic factors. We investigated these relationships using nationally standardized exam results at ages 11 and 14 from a UK longitudinal cohort study. We found that teacher expectation of achievement was strongly correlated with educational test scores. Furthermore, the accuracy of teacher expectation was patterned by pupil socioeconomic background but not teacher characteristics. The accuracy of teacher expectation related to pupil’s genetic liability to education as captured by a polygenic score for educational attainment. Despite correlation with the polygenic score, we found no strong evidence for genomewide SNP heritability in teacher reporting accuracy.

## Introduction

Teachers’ expectations of ability can affect pupils’ academic achievement throughout their educational career from initial enrolment through to the end of compulsory schooling^1–6^. They can influence the subjects that pupils take, whether they are entered into an advanced stream, the level of exam they are entered to, how long they remain in education and ultimately their educational attainment^7–10^. These expectations are based on a teacher’s understanding and experience with pupils over an extended period and can have advantages over pupil achievement measured by test performance^11,12^. For example, expectations may avoid a misleading representation of a pupil’s ability if they tested on a particularly good or bad day, they avoid incentives to “teach to the test”, they may remove the stress of formalised testing, and they can ensure that ability is measured using a broader range of factors than test performance alone^13–15^.

However, disadvantages also exist with teacher expectations. First, there is potential for either conscious or unconscious bias against specific pupils or groups, such as by gender, socioeconomic background, ethnicity or special educational needs status^6,10,16–20^. Teacher expectation theory posits that while teachers form inferences about their student’s future academic achievement for individuals and groups of students, their expectations of pupils may be biased by students’ backgrounds^21–23^. Second, variation in teacher and classroom characteristics may result in systematic differences in the accuracy of teacher expectations. Teachers with larger class sizes have less individual contact time with each pupil, meaning that their expectations may be less reliable than teachers with smaller classes^24^. Third, teachers may only have a small sample of previous students to draw upon so the accuracy of their expectations of future pupil performance may be dependent on their level of experience^25^. Given these advantages and disadvantages, teacher expectations can be used for early assessment and streaming before being replaced by formalised testing and assessment later in schooling. However, recent policy updates highlight how this is not always the case. The Covid-19 pandemic led to the use of assessments from teacher expectations for determining academic performance in the UK, following heavy criticism of the UK Government’s initial statistical model for exam results which was reported to have widespread inconsistencies.

Previous studies have shown that systematic differences exist in the accuracy of teacher expectations of subsequent achievement across groups of pupils. On average teachers underestimate outcome for students with special educational needs, those of black-African and black-Caribbean ethnic origin, and those of a lower socioeconomic position, and boys^16,17,26–28^. For example, a meta-analysis of 39 studies demonstrated that teacher’s expectations of pupils was linked to pupils’ ethnicity, with higher expectations being held for European-American pupils than for ethnic-minority pupils^29^. These systematic differences can be detrimental to pupils who are under or overestimated by their teachers. For example, pupils who felt undervalued by teachers may be less likely to be engaged in school and have lower achievement than expected^30^. Conversely, pupils whose ability is overestimated may be overlooked by teachers or placed into streams that are too advanced for them and they therefore may not receive adequate support to accomplish their academic potential^1,9,16^.

New data and methods offer the opportunity to examine the accuracy of teacher expectations in novel ways. The use of genetic data in educational research is growing and there is now evidence that many genetic variants (single nucleotide polymorphisms, SNPs) associate with educational attainment, achievement and progress^31–33^. The largest genome-wide association study of education to date identified over 1000 SNPs which combined into a polygenic score (PGS) explain around 12% of the variation in educational attainment. Because genetic variation is set at birth and cannot be affected by environmental factors post-conception, associations between genetic factors and individual characteristics are robust to confounding and reverse causation that pervade much educational research^34^. While genetic variation is not directly observable, its effects on education are.

If teachers’ expectations of their students’ achievement are unbiased, then the accuracy of their expectations of student attainment should not be related to the students’ polygenic scores for educational attainment. Alternatively, an associations between the teachers’ expectations of achievement may suggest bias towards other unmeasured characteristics. For example, if teachers overestimate the achievement of a given group (for example girls from high socioeconomic position (SEP) backgrounds) and underestimate the achievement of another group (for example boys from low SEP backgrounds) then we might expect the difference between teacher expectations and exam results to be partially explained by the educational attainment polygenic score. To investigate this, we estimated the association of teacher expectation accuracy with socioeconomic, demographic and genetic factors in the Avon Longitudinal Study of Parents and Children (ALSPAC), a UK longitudinal cohort.

We investigated the following three hypotheses: (1) do teacher expectations accurately associate with realised achievement?, (2) do teacher expectations associate with teacher characteristics and pupil socioeconomic and demographic factors? and (3) Do pupils’ common genetic variation explain differences in the accuracy of teacher expectations?

## Results

Due to attrition and item non-response in the ALSPAC cohort, the complete case samples of ALSPAC participants available for analyses are 2341 at Key Stage 2 (age 11) and 3696 at Key Stage 3 (age 14). We therefore ran multiple imputation to recover missing data and increase the statistical power of our analyses. Our multiple imputation sample was 7465, with imputations run over 100 iterations. Teacher expectation accuracy was obtained by regressing realised achievement in standardised national examinations for Mathematics, English and Science on teacher expectations of performance in these subjects.

### Association of teacher expectations and achievement

Each one standard deviation (SD) increase in teacher expectation was associated with a 0.88 (95% CI 0.87, 0.89) and 0.92 (95% CI 0.91, 0.93) SD increase in realised achievement at Key Stages 2 and 3 respectively in the imputed data (Table 1). Teacher expectations explained a large amount of variation in realised pupil achievement as demonstrated by the high R^2^ values of 0.78 and 0.85 at Key Stages 2 and 3 respectively.

**Table 1:** Standardised association between achievement and teacher expectations.

	Effect estimate (95% CI)		R^2^	
	Complete case	Imputed	Complete case	Imputed
Key Stage 2 (age 11)	0.76 (0.74, 0.78)	0.88 (0.87, 0.89)	0.76	0.78
Key Stage 3 (age 14)	0.83 (0.82, 0.84)	0.92 (0.91, 0.93)	0.85	0.85

### Phenotypic predictors of teacher expectation accuracy

Teacher expectation accuracy associated with some demographic and socioeconomic measures at both Key Stages (Table 2). At Key Stage 2 pupils with less educated mothers underperformed their teacher’s expectations relative to pupils with degree educated mothers (O-level: − 0.13, 95% CI − 0.23, − 0.034; Vocational: − 0.29, 95% CI − 0.41, − 0.16; CSE: − 0.38, 95% CI − 0.49, − 0.26). Children of parents from lower socioeconomic backgrounds underperformed their teacher’s expectations relative to pupils whose parents were from the highest socioeconomic background (II: − 0.11, 95% CI − 0.20, − 0.015; III non-manual: − 0.17, 95% CI − 0.28, − 0.07; III manual: − 0.22, 95% CI − 0.34, − 0.10; IV: − 0.34; 95% CI − 0.50, − 0.18). Pupils born later in the school year slightly underperformed compared to those who were born earlier in the year (− 0.011, 95% CI − 0.018, − 0.005). Associations were consistent at Key Stage 3 for maternal education (O-level: − 0.16, 95% CI − 0.28, − 0.04; Vocational: − 0.21, 95% CI − 0.35, − 0.08; CSE: − 0.28, 95% CI − 0.41, − 0.15) and month of birth (− 0.013, 95% CI − 0.021, − 0.005), but not for parental social class. Children from families in the lowest two income categories also underperformed teacher expectations at Key Stage 3 compared to those in the top category £100–199 per week: − 0.12; 95% CI − 0.23, − 0.005; Less than £100 per week: − 0.17; 95% CI − 0.33, − 0.018). There was little evidence children of differing sex or SEN status under/overperformed their teacher’s expectations.

**Table 2:** Associations between teacher expectation, socioeconomic and demographic variables.

	Teacher expectation accuracy at Key Stage 2 (age 11)		Teacher expectation accuracy at Key Stage 3 (age 14)	
	Coefficient (95% CI)	P value	Coefficient (95% CI)	P value
**Gender**				
Female	*Reference*		*Reference*	
Male	0.010 (− 0.04, 0.06)	0.710	0.024 (− 0.036, 0.08)	0.433
Month of delivery^a^	− 0.011 (− 0.018, − 0.005)	0.001	− 0.013 (− 0.021, − 0.005)	0.002
**SEN status**				
Not statemented	*Reference*		*Reference*	
Has a statement	− 0.00024 (− 0.23, 0.23)	0.998	− 0.09 (− 0.35, 0.17)	0.502
**Mothers’ highest education**				
Degree	*Reference*		*Reference*	
A level	− 0.09 (− 0.18, 0.0017)	0.054	− 0.04 (− 0.16, 0.07)	0.438
O level	− 0.13 (− 0.23, − 0.034)	0.008	− 0.16 (− 0.28, − 0.04)	0.007
Vocational	− 0.29 (− 0.41, − 0.16)	< 0.001	− 0.21 (− 0.35, − 0.08)	0.002
CSE	− 0.38 (− 0.49, − 0.26)	< 0.001	− 0.28 (− 0.41, − 0.15)	< 0.001
**Parental social class**				
I	*Reference*		*Reference*	
II	− 0.11 (− 0.20, − 0.015)	0.023	0.08 (− 0.014, 0.18)	0.096
III non-manual	− 0.17 (− 0.28, − 0.07)	0.001	0.05 (− 0.06, 0.17)	0.379
III manual	− 0.22 (− 0.34, − 0.10)	< 0.001	0.021 (− 0.11, 0.15)	0.751
IV	− 0.34 (− 0.50, − 0.18)	< 0.001	0.028 (− 0.15, 0.21)	0.764
V	− 0.018 (− 0.37, 0.33)	0.919	0.11 (− 0.31, 0.53)	0.600
**Income, £ per week**				
Over 400	*Reference*		*Reference*	
300–399	− 0.019 (− 0.10, 0.06)	0.637	0.025 (− 0.07, 0.12)	0.602
200–299	− 0.039 (− 0.12, 0.04)	0.342	− 0.025 (− 0.12, 0.07)	0.595
100–199	− 0.07 (− 0.18, 0.028)	0.153	− 0.12 (− 0.23, − 0.005)	0.041
Less than 100	− 0.032 (− 0.17, 0.10)	0.643	− 0.17 (− 0.33, − 0.018)	0.028
**Teacher gender**^**b**^				
Female				
Male	− 0.039 (− 0.12, 0.040)	0.335		
**Length of teaching time**^**b**^				
10 + years				
3–9 years	0.038 (− 0.031, 0.11)	0.285		
1–2 years	0.10 (− 0.06, 0.26)	0.230		
Less than 1 year	0.07 (− 0.15, 0.29)	0.534		
Class size^b^, per additional 10 pupils	− 0.025 (− 0.10, 0.05)	0.532		
Constant	0.44 (0.20, 0.68)	< 0.001	0.18 (0.07, 0.29)	0.002

Positive values reflect pupils who overperformed relative to their teacher’s expectations, while negative values reflect pupils who underperformed relative to their teacher’s expectations. Results for complete case analyses presented in Supplementary Table S1.

^a^Where September = 1, October = 2 etc. to reflect the month of entry in UK schooling.

^b^Data available for KS2 only.

There was little evidence that teacher gender (− 0.039, 95% CI − 0.12, 0.40), teacher experience (e.g. 1–2 years vs 10 or more years: 0.07, 95% CI − 0.15, 0.29), or class size (− 0.025, 95% CI − 0.10, 0.05 per additional 10 pupils) associated with teacher expectation accuracy. Results were consistent across the imputed and complete case analyses (Supplementary Table S1).

### Genotypic predictors of teacher expectation accuracy

We estimated the association between teacher expectation accuracy and a pupil’s polygenic score for educational attainment built from the largest GWAS of education to date^32^. Pupil’s educational attainment polygenic score associated with teacher expectation accuracy at both Key Stage 2 (0.13; 95% CI 0.11, 0.16) and Key Stage 3 (0.10; 95% CI 0.08, 0.13) (Table 3). These associations persisted after adjustment for demographic and socioeconomic factors, and the first 20 principal components of ancestry (KS2: 0.08; 95% CI 0.06, 0.11; KS3: 0.07; 95% CI 0.04, 0.10). This suggests that pupils with higher polygenic scores for educational attainment were more likely to outperform their teachers’ expectations than children with lower polygenic scores.

**Table 3:** Associations between teacher expectation and pupil’s polygenic scores (PGS).

	Teacher expectation accuracy at Key Stage 2 (age 11)		Teacher expectation accuracy at Key Stage 3 (age 14)	
	Coefficient (95% CI)	P-value	Coefficient (95% CI)	P-value
PGS only	0.13 (0.11, 0.16)	< 0.001	0.10 (0.08, 0.13)	< 0.001
PGS adjusted for covariates	0.08 (0.06, 0.11)	< 0.001	0.07 (0.04, 0.10)	< 0.001

Positive values reflect pupils who overperformed relative to their teacher’s expectations, while negative values reflect pupils who underperformed relative to their teacher’s expectations. n = 7465. Full results in Supplementary Table S2. Results for complete case analyses presented in Supplementary Table S3.

### SNP heritability of teacher expectation accuracy

**Figure 1:**
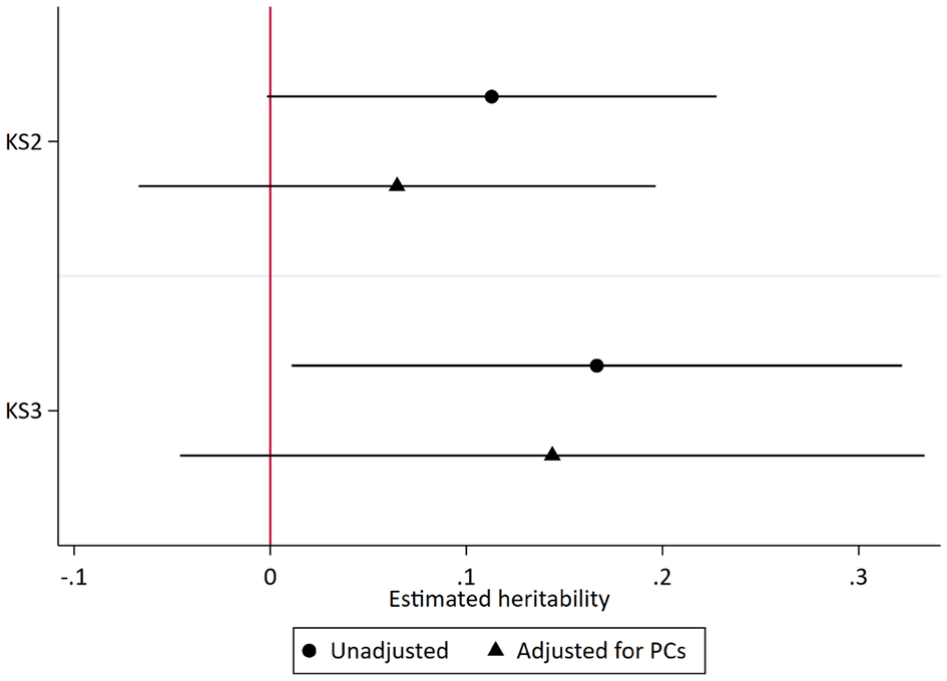
Estimated SNP heritability of teacher expectation accuracy at Key Stage 2 (age 11) and Key Stage 3 (age 14). Models control for the first 20 principal components of population structure.

We used GCTA-GREML^35^ to assess SNP heritability of teacher expectation accuracy. We found weak evidence for SNP heritability of teacher expectation accuracy at both ages. SNP heritability was estimated at 6.5% (95% CI − 6.7%, 19.6%) for Key Stage 2 and 14% for Key Stage 3 (95% CI − 4.6%, 33.4%) after adjustment for principal components of ancestry. Like the PGS results, this suggests that pupil’s under or over-performance relative to their teacher’s expectations may relate to their genome-wide genetic variation (Fig. 1).

## Discussion

Our results suggested that teachers’ expectation of their pupil’s achievement was generally accurate at two Key Stages of UK education (ages 11 and 14). We found evidence that teacher expectation accuracy was related to some socioeconomic or demographic factors, principally pupil’s maternal education and age in year at both Key Stages, and parental social class and household income at Key Stage 2 and 3 respectively. These patterns were consistently in the same direction, whereby pupil’s from more disadvantaged backgrounds underperformed compared to their advantaged peers. Our findings conform to those from previous studies which have found differential teacher expectation accuracy towards certain groups of pupils such as those from lower socioeconomic position backgrounds^3,6,10,16,27,28,36–38^. For example, disparity was found between teacher assessed measures and Foundation Stage Profile assessment on socioeconomic and demographic factors including income, gender, special educational needs status and ethnicity^4^. We found little evidence that teacher gender, teacher experience or class size were associated with the accuracy of teacher expectations. This contrasts to previous research that has observed strong associations between these factors and the accuracy of teacher expectations^16,25^.

We found mixed evidence for associations between genetic factors and teacher expectation accuracy. Pupils with a higher value of the polygenic score from a large GWAS of educational attainment^32^ were more likely to outperform their teachers’ expectations compared to children with lower polygenic scores. Using all genomewide data within a GREML-GCTA framework we found only weak evidence for SNP heritability at both ages, though these results should be interpreted with caution due to their imprecision. These results suggest that some of the variation in teacher reporting accuracy can be explained by genetic variation at the pupil level. Conversely, they suggest that most of the variation in teacher expectation accuracy can be explained by non-genetic (environmental and residual) factors. Genetic liability towards educational performance could operate through a range of mediating mechanisms, such as personality characteristics or attitudes to learning and schoolwork^16,36^. In this way, ‘invisible’ genetic variation may become visible to teachers, influencing their expectations of a pupil’s future performance. Future studies with larger sample sizes are required to verify these findings, however our results build upon previous studies demonstrating robust associations between genetic factors and achievement throughout schooling^31,39,40^.

Our analyses were unable to determine whether inaccuracies in teacher expectation were due to error or detrimental bias on the teachers part, i.e. whether teachers were prejudiced against specific groups of pupils^2,41^. Prejudice on the teachers part however may have been more likely to result in pupils *over* performing their teachers (unfairly negative) expectations. Similarly, pupil behavioural change due to self-fulfilling prophecies from inaccurate teacher forecasts may be expected to result in accurate teacher expectations, even if they are prejudiced^42^. Regardless, our findings highlight that some groups of students systematically underperform their teachers’ expectations. We were unable to reliably investigate how ethnicity related to teacher expectations because the ALSPAC cohort had low numbers of ethnic minority participants. Previous studies conducted on more ethnically representative cohorts have shown that teacher expectations differ by pupil ethnicity^6,18,30^.

Several limitations exist with this study. First, generalisability of these findings to the wider UK school population may be limited. Our sample was ethnically homogenous and restricted to those who were recruited from a single geographical area over a three school-year period. This tightly defined sampling frame means that there will likely be reduced environmental and genetic variation in our sample compared to the broader population of the UK. Furthermore, within ALSPAC there is greater attrition for pupils from families of lower socioeconomic position and poorer general health^43^, meaning that this demographic are underrepresented and our complete case analyses may be biased. Results from our multiple imputation analyses were broadly consistent with the complete case analyses, suggesting that bias due to attrition may be limited.

Second, the age of the sample may also limit the generalisability of our findings. We examined the accuracy of teacher expectations at ages 11 and 14, but our results may not be transportable to earlier of later stages of education. Additionally, the participants were educated between 2001 and 2006 and teacher expectations may have change since this period.

Third, the accuracy of teacher expectations may have differed across Maths, English and Science subjects as many participants will have had subject specific teachers. Our decision to combine across these subjects was taken to provide a more accurate measure of the pupil’s overall academic performance and reflect any general teacher expectation bias. Furthermore, teacher expectations of pupil achievement were provided as categorical levels for each subject, meaning that there was reduced variation in this measure when compared to point scores used for assessment.

Fourth, many variables were subject to potential measurement error. For example, family socioeconomic position is a complex construct encompassing education, income, wealth and other factors, yet we were only able to proxy this using weekly household income, parental social class and the highest level of maternal education^44,45^. To improve statistical power, we leveraged the larger sample of responses from study mother’s reports of their partners occupation and income, but it is likely that the mother reports will have contained greater measurement error than direct partner reports. Additionally, there was strong correlation between the teacher predicted scores and pupil achieved test scores, even though only a coarse measure of predicted achievement was available. As such, the discrepancies between predicted and actual scores are likely to reflect some measurement error. However, this measurement error may not have differed by the pupils’ other characteristics.

Finally, because of the ethnic homogeneity of the ALSPAC sample, the European-centric focus of genetic studies, and the need to exclude non-Europeans due to systematic ancestry differences arising from population stratification (which can induce spurious genotype–phenotype associations)^46–48^, we were only able to perform the analyses amongst white participants of European ancestry. Previous studies have demonstrated that the direction of teacher expectation accuracy varies by ethnicity^4^. However, previous work has demonstrated that trait-associated genetic markers do not perform well across ancestral groups^49^. Larger genotyped samples of ethnic minorities are therefore required to explore this issue further.

In conclusion, this study investigated potential patterns of teacher expectation accuracy by socioeconomic, demographic and genetic factors. We found evidence of systematic socioeconomic and genetic patterning in teacher expectations. Pupils from more disadvantaged backgrounds underperformed their teachers’ expectations compared to their more advantaged peers, and those with higher genetic liability for educational attainment outperformed their teachers’ expectations relative to pupils with lower genetic liability for educational attainment.

## Methods

### Study participants

We used data from the Avon Longitudinal Study of Parents and Children (ALSPAC), a longitudinal birth cohort study based in Bristol, UK. ALSPAC initially recruited 14,541 pregnant women with an expected delivery date between April 1991 and December 1992. When the oldest children were approximately seven years of age, an attempt was made to recruit eligible children that were not included in the original sample. This resulted in a total eligible sample size of 15,454 pregnancies of which 14,901 children were alive at one year of age. For full cohort details and study design see^43,50^. The ALSPAC cohort was representative of the UK population in 1991 on many criteria, but had underrepresentation of ethnic minorities, single parent families and those of lower socio-economic position. Ethical approval for the study was obtained by the ALSPAC Ethics and Law Committee (ALEC) and the Local Research Ethics Committees. Informed consent for the use of data collected via questionnaires and direct assessments was obtained from participants following the recommendations of the ALEC. Informed consent was provided by the study mothers when the participants were minors. All methods were carried out in accordance with relevant guidelines and regulations. Questionnaires were completed by study mothers, the child’s schoolteachers and headteachers to obtain information relating to family background and the school/classroom. The study website contains details of all the data that is available through a fully searchable data dictionary and variable search tool (see http://www.bristol.ac.uk/alspac/researchers/our-data/). Due to low numbers in the minority ethnicity groups and problems with multi-ancestry genetic analyses (below), all non-White participants were excluded.

### Educational outcomes

#### Realised achievement

We used fine graded achievement scores at two of the major “Key Stages” of UK education (Key Stage 2, at ages 7–11, Key Stage 3, at ages 11–14) obtained from the UK National Pupil Database (NPD) through data linkage to the ALSPAC cohort. Achievement scores were determined from examinations at the end of each Key Stage.

#### Teacher expected achievement

Teacher expected achievement was available from the NPD as categorical variables indicating the national curriculum level (i.e. 3, 4, 5) that a pupil was expected to achieve for each of Mathematics, English and Science. The average of these three measures was taken and converted to a point score to enable comparability with achievement scores (Supplementary Table S4). All achievement scores were rounded for comparability.

#### Accuracy of teacher expectations

To determine the accuracy of teacher expectations we used residuals from a regression of realised achievement (the dependent variable) on teacher expectation scores (the independent variable) at Key Stages 2 and 3. A positive value therefore indicates that a pupil outperformed their teacher’s expectation in their examinations. For ease of interpretation, accuracy scores were standardised to follow a normal distribution with mean zero and standard deviation one. This enabled investigation into systematic inaccuracy of teacher expectations against a variety of demographic and teacher characteristics, and exploration of heterogeneity in the teacher expectations across groups.

### Covariates

Information on participant sex at birth and month of birth was obtained from birth records. Month of birth was recoded with September as the first month to represent age in school year (the school year in the UK starts in September). Self-report questionnaires completed by the study mothers during pregnancy provided information on family socioeconomic position. Socioeconomic position was proxied by parental social class based on occupation at cohort member birth, the mother’s highest education qualification at cohort member birth, and family income at cohort member age four. Study mothers reported their own and their partners’ occupation, with responses coded to the Standard Occupational Classification (SOC) codes and converted to social class based on occupation, with the following seven bands: I (Professional occupations); II (Managerial and technical occupations); III-NM (Skilled non-manual occupations); III-M (Skilled manual occupations); IV (Partly-skilled occupations); V (Unskilled occupations); Armed forces. Armed forces responses to social class were recoded to II due to low number of observations. Where both maternal and paternal social class were available, the highest social class was taken. Mothers highest level of education was categorised as follows: Degree; A-level (a post-compulsory qualification at age 18); O-level (a subject based academic qualification at age 16); Vocational qualifications; Certificate of Secondary Education (CSE, a general qualification at age 16). Family income per week was reported in the following bands: less than £100; £100–199; £200–299; £300–399; £400 or more; Don’t know. Responses of “don’t know” were coded as missing.

Teachers provided information through self-report questionnaires at Key Stage 2 (age 11) about their gender (categorised as “Male” or “Female”), length of service (less than one year, 1–2 years, 3- years, 10 + years), and the number of pupils per class. School headteachers provided information on the Special Educational Needs (SEN) status of participants, characterised as: (1) Has a statement; (2) Currently being assessed; (3) Not statemented; (4) Has been refused. SEN status of 2, 3 and 4 were re-coded to “Not statemented”.

### Genotyping, quality control and imputation

DNA of the ALSPAC children was extracted from blood, cell line and mouthwash samples, then genotyped using reference panels and subjected to standard quality control approaches. ALSPAC children were genotyped using the Illumina HumanHap550 quad chip genotyping platforms by 23andme subcontracting the Wellcome Trust Sanger Institute, Cambridge, UK and the Laboratory Corporation of America, Burlington, NC, US. Standard quality control methods were applied to the resulting genome-wise data. Individuals were excluded based on sex-mismatch, minimal or excessive heterozygosity (< 0.320 and > 0.345), individual missingness greater than 3% and insufficient sample reduction (IBD < 0.8). Multidimensional scaling was used to stratify the population, comparing with Hapmap II (release 22) European descent (CEU), Han Chinese, Japanese and Yoruba reference populations; all individuals with non-European ancestry were removed. SNPs with a minor allele frequency (MAF) of less than 1%, a call rate of less than 95% or evidence for violations of Hardy–Weinberg equilibrium (p-value < 5 × 10^–7^) were removed. Cryptic relatedness was measured as a proportion of identity-by-descent (IBD > 0.1), with related participants passing all other quality control thresholds retained in subsequent phasing and imputation, described fully in supporting information. 8237 children passed these quality control filters.

Children's genotypes were jointly phased and imputed with the genotypes of the ALSPAC mothers (Illumina human660W quad (mothers)), combining 477,482 SNP genotypes which were in common between the samples. SNPs with genotype missingness above 1% were removed due to poor quality (11,396 SNPs removed) and a further 321 participants due to potential ID mismatches. This resulted in a dataset of 17,842 participants containing 465,740 SNPs (112 removed during liftover and 234 were out of Hardy Weinberg Equilibrium after combination). Haplotypes were estimated using ShapeIT (v2.r644), utilizing relatedness during phasing. A phased version of the 1000 genomes reference panel (Phase 1, Version 3) from the Impute2 reference data repository (phased using ShapeIT v2.r644, haplotype release data Dec 2013) was obtained. Imputation of the target data was performed using Impute V2.2.2 against the reference panel (all polymorphic SNPs excluding singletons), using all 2186 reference haplotypes (including non-Europeans). This gave 8237 eligible children and 8196 eligible mothers with available genotype data after exclusion of related participants using cryptic relatedness measures described previously.

### Polygenic scores

A PGS was generated from the largest GWAS of educational attainment. ALSPAC participants were excluded from the meta-analysis used to generate the PGS to reduce bias due to overfitting alongside 23andMe participants due to data sharing agreements. The PGS was created using the software package PRSice^51^ using all SNPs that were identified to associate with years of education. The scores were calculated as a weighted sum of educational attainment associated SNPs weighted by their effect size. SNPs were clumped and the SNP with the smallest P-value in each 250 kb window was retained. All other SNPs in linkage disequilibrium with an r^2^ > 0.1 were removed.

### Multiple imputation

Due to attrition and item non-response, 2341 and 3696 participants had missing data on at least one variable at KS2 and KS3 respectively (Supplementary Fig. S1). To increase statistical power and reduce potential selection bias within primary findings due to attrition^52^, we conducted multiple imputation by chained equations (MICE)^53^ under the assumption that data were missing at random (MAR) conditional upon the data included in the imputation model^54^. To help overcome this assumption, additional covariates were included as predictors in the imputed dataset to incorporate additional information and improve imputation accuracy^53,55,56^. MICE is a method based on data augmentation, iteratively estimating parameters for the distribution of each variable, and using this to predict the missing values. All variables except for genotype were imputed and variables that did not follow a normal distribution were transformed. Imputation was conducted in Stata 16 using the *mim* command^57,58^. A total of 100 imputed datasets were generated, and the results were pooled for each regression analyses. We only imputed phenotypic data using this approach (see “Genotyping, quality control and imputation” for imputation of genetic data). The pooled imputed dataset contained 7465 participants, of whom all had genetic data. We found little evidence that distributions of the imputed and observed values differed (Table 4). While the proportion of missing data were large in some cases, previous research has demonstrated that this will not bias imputation results and is not a reliable guide for comparing the accuracy between complete case and multiple imputation analyses^59^. Table 1 displays the distributions of key variables across the complete case and multiple imputation samples.

**Table 4:** Complete case and imputed summary statistics.

Variable	Complete case dataset		Imputed dataset^c^	
	N	Mean (SD)	N	Mean (SD)
**Gender**	5499		7465	
Male	2780	50.55	3810	51.04
Female	2719	49.45	3655	48.96
Month of delivery^a^	5499	6.65 (3.72)	7465	6.63 (3.74)
**SEN status**	5499		7465	
Has a statement	64	1.16	7368	98.70
Not statemented	5435	98.84	97	1.30
**Mothers highest education**	5499		7465	
Degree	940	17.09	1131	15.14
A level	1451	26.39	1863	24.95
O level	1977	35.95	2663	35.68
Vocational	460	8.37	692	9.28
CSE	671	12.20	1116	14.95
**Parental social class**	5499		7465	
I	868	15.78	1088	14.57
II	2457	44.68	3247	43.51
III non-manual	1374	24.99	1891	25.32
III manual	585	10.64	880	11.80
IV	191	3.47	316	4.22
V	24	0.44	43	0.58
**Family income per week**	5499		7465	
Less than £100	270	4.91	436	5.88
£100–£199	710	12.91	1040	13.92
£200–£299	1498	27.24	2011	26.94
£300–£399	1306	23.75	1745	23.31
More than £400	1715	31.19	2233	29.95
Number of pupils on class register^b^	2914	2.83 (0.51)	7465	2.82 (0.50)
**Teacher gender**^**b**^	2898		7465	
Male	728	25.12	1874	25.12
Female	2170	74.88	5591	74.88
**Length of service**^**b**^	2580		7465	
Less than 1 year	48	1.65	123	1.64
1–2 years	110	3.79	264	3.57
3–9 years	1156	39.85	2933	39.31
10 or more years	1587	54.71	4145	55.49

Imputed summary statistics calculated from 100 imputed datasets.

^a^Where September = 1, October = 2 etc. to reflect the month of entry in UK schooling.

^b^Information available for KS2 only.

^c^Summary statistics calculated from 100 imputed datasets.

### Statistical analysis

We estimated associations between realised achievement and teacher expectations, expectation accuracy and teacher characteristics, and expectation accuracy and polygenic scores using linear regression. We performed separate analyses for Key Stages 2 and 3 to assess teacher expectation accuracy at ages 11 and 14.

We estimated the SNP heritability of the teacher expectation accuracy using genome-wide complex trait analysis (GCTA) with genomic-relatedness-based restricted maximum-likelihood estimation (GREML)^35^. SNP heritability is defined as the proportion of total variation in a phenotype (the teacher expectation accuracy) that can be explained by common genetic variation in all measured SNPs^35^, akin to a correlation coefficient^60^. GCTA first estimates the genetic similarity between every pair of unrelated individuals using measured variation across the genome, and compares this similarity with the phenotypic similarity of each pair. If more genetically similar pairs are more phenotypically similar than genetically dissimilar pairs, then the heritability estimate for a phenotype will be higher.

For all genotypic analyses we make two further sample restrictions. First, we restrict the sample to unrelated participants in ALSPAC (less related than 2nd cousins) as indicated by their genotypic similarity. Second, we restrict our sample to participants of European ancestry only due to poor polygenic score performance in diverse ancestral groups. Further to these selections, we control for the first 20 principal components of population structure in all genotypic analyses to reduce population stratification bias.

## Supplementary Information

Supplementary Information.

## Data Availability

The empirical dataset has been archived with the ALSPAC study under the project identifier B2193 and will be made available to individuals who obtain the necessary permissions from the study’s executive committee.

## Code availability

All code used to process and analyse the data are available at https://github.com/Ciarrah/tchr_rprtng_ccrcy/.
