# Supplementary material for: Investigating how the accuracy of teacher expectations of pupil performance relate to socioeconomic and genetic factors
Source: Sci Rep. 2022 May 3;12:7120. doi: 10.1038/s41598-022-11347-w (PMC9065134; doi:10.1038/s41598-022-11347-w)

# Supplementary material

### Table S1: Associations between teacher expectations, socioeconomic and demographic variables in complete case sample. Positive values reflect pupils who overperformed relative to their teacher’s expectations, while negative values reflect pupils who underperformed relative to their teacher’s expectations.

|  | **Teacher expectation accuracy at Key Stage 2 (age 11)** | | **Teacher expectation accuracy at Key Stage 3 (age 14)** | |
| --- | --- | --- | --- | --- |
|  | **Coefficient (95% CI)** | **P value** | **Coefficient (95% CI)** | **P value** |
| **Gender** |  |  |  |  |
| *Female* | *Reference* |  | *Reference* |  |
| *Male* | 0.022 (-0.06, 0.10) | 0.595 | 0.036 (-0.035, 0.11) | 0.321 |
| **Month of delivery^1^** | -0.012 (-0.022, -0.0008) | 0.036 | -0.016 (-0.026, -0.006) | 0.002 |
| **SEN status** |  |  |  |  |
| *Not statemented* | *Reference* |  | *Reference* |  |
| *Has a statement* | -0.06 (-0.35, 0.24) | 0.707 | -0.04 (-0.37, 0.28) | 0.796 |
| **Mothers highest education** |  |  |  |  |
| *Degree* | *Reference* |  | *Reference* |  |
| *A level* | -0.14 (-0.29, 0.007) | 0.062 | -0.09 (-0.22, 0.034) | 0.153 |
| *O level* | -0.11 (-0.26, 0.038) | 0.144 | -0.19 (-0.32, -0.06) | 0.004 |
| *Vocational* | -0.27 (-0.46, -0.08) | 0.006 | -0.23 (-0.40, -0.07) | 0.006 |
| *CSE* | -0.34 (-0.52, -0.16) | <0.001 | -0.31 (-0.47, -0.16) | <0.001 |
| **Parental social class** |  |  |  |  |
| *I* | *Reference* |  | *Reference* |  |
| *II* | -0.12 (-0.25, 0.020) | 0.095 | 0.07 (-0.05, 0.19) | 0.239 |
| *III non-manual* | -0.17 (-0.33, -0.019) | 0.028 | 0.08 (-0.06, 0.22) | 0.272 |
| *III manual* | -0.28 (-0.47, -0.10) | 0.002 | -0.013 (-0.17, 0.15) | 0.874 |
| *IV* | -0.33 (-0.59, -0.08) | 0.010 | -0.04 (-0.26, 0.18) | 0.739 |
| *V* | 0.025 (-0.51, 0.56) | 0.928 | -0.06 (-0.61, 0.50) | 0.834 |
| **Income, £ per week^2^** |  |  |  |  |
| *Over 400* | *Reference* |  | *Reference* |  |
| *300-399* | -0.0033 (-0.12, 0.11) | 0.956 | 0.09 (-0.09, 0.28) | 0.325 |
| *200-299* | -0.06 (-0.18, 0.06) | 0.303 | 0.18 (0.011, 0.36) | 0.037 |
| *100-199* | -0.13 (-0.28, 0.016) | 0.080 | 0.26 (0.09, 0.44) | 0.004 |
| *Less than 100* | -0.12 (-0.33, 0.09) | 0.256 | 0.23 (0.05, 0.41) | 0.014 |
| **Teacher gender^2^** |  |  |  |  |
| *Female* | *Reference* |  |  |  |
| *Male* | -0.027 (-0.12, 0.07) | 0.579 |  |  |
| **Length of teaching time^2^** |  |  |  |  |
| *10+ years* | *Reference* |  |  |  |
| *3-9 years* | 0.05 (-0.033, 0.13) | 0.237 |  |  |
| *1-2 years* | 0.11 (-0.10, 0.31) | 0.306 |  |  |
| *Less than 1 year* | 0.09 (-0.21, 0.39) | 0.547 |  |  |
| **Class size^2^, per additional 10 pupils** | -0.05 (-0.14, 0.05) | 0.313 |  |  |
| **Constant** | 0.53 (0.21, 0.85) | 0.001 | 0.022 (-0.20, 0.25) | 0.849 |

*^1^ Where September = 1, October = 2 etc to reflect the month of entry in UK schooling.^2^ Data available for KS2 only*

### Table S2: Associations between teacher expectation and pupil’s polygenic scores (PGS) in multiple imputation sample. Positive values reflect pupils who overperformed relative to their teacher’s expectations, while negative values reflect pupils who underperformed relative to their teacher’s expectations. n=7,465.

|  | Teacher expectation accuracy at Key Stage 2 (age 11) | | Teacher expectation accuracy at Key Stage 3 (age 14) | |
| --- | --- | --- | --- | --- |
|  | Coefficient (95% CI) | P value | Coefficient (95% CI) | P value |
| **PGS** | 0.08 (0.06, 0.11) | <0.001 | 0.07 (0.04, 0.10) | <0.001 |
| **Gender** |  |  |  |  |
| *Female* | *Reference* |  | *Reference* |  |
| *Male* | 0.007 (-0.04, 0.06) | 0.782 | 0.022 (-0.038, 0.08) | 0.477 |
| **Month of delivery^1^** | -0.012 (-0.019, -0.005) | 0.001 | -0.013 (-0.021, -0.005) | 0.002 |
| **SEN status** |  |  |  |  |
| *Not statemented* | *Reference* |  | *Reference* |  |
| *Has a statement* | 0.027 (-0.20, 0.25) | 0.812 | -0.07 (-0.33, 0.19) | 0.585 |
| **Mothers’ highest education** |  |  |  |  |
| *Degree* | *Reference* |  | *Reference* |  |
| *A level* | -0.06 (-0.15, 0.04) | 0.225 | -0.016 (-0.13, 0.10) | 0.785 |
| *O level* | -0.08 (-0.18, 0.035) | 0.088 | -0.12 (-0.24, -0.009) | 0.048 |
| *Vocational* | -0.22 (-0.35, -0.10) | <0.001 | -0.16 (-0.29, -0.020) | 0.025 |
| *CSE* | -0.31 (-0.43, -0.20) | <0.001 | -0.22 (-0.35, -0.09) | 0.001 |
| **Parental social class** |  |  |  |  |
| *I* | *Reference* |  | *Reference* |  |
| *II* | -0.09 (-0.18, 0.0016) | 0.054 | 0.09 (-0.0013, 0.19) | 0.053 |
| *III non-manual* | -0.15 (-0.26, -0.05) | 0.005 | 0.07 (-0.04, 0.19) | 0.220 |
| *III manual* | -0.19 (-0.31, -0.07) | 0.002 | 0.05 (-0.08, 0.18) | 0.467 |
| *IV* | -0.32 (-0.48, -0.16) | <0.001 | 0.05 (-0.14, 0.23) | 0.615 |
| *V* | 0.030 (-0.32, 0.38) | 0.868 | 0.16 (-0.26, 0.58) | 0.467 |
| **Income, £ per week** |  |  |  |  |
| *Over 400* | *Reference* |  | *Reference* |  |
| *300-399* | -0.017 (-0.10, 0.06) | 0.678 | 0.028 (-0.06, 0.12) | 0.551 |
| *200-299* | -0.032 (-0.11, 0.05) | 0.437 | -0.017 (-0.11, 0.08) | 0.716 |
| *100-199* | -0.06 (-0.16, 0.04) | 0.249 | -0.10 (-0.22, 0.011) | 0.077 |
| *Less than 100* | -0.007 (-0.14, 0.13) | 0.915 | -0.15 (-0.31, 0.0045) | 0.057 |
| **Constant** | 0.32 (0.22, 0.41) | <0.001 | 0.12 (0.0021, 0.24) | 0.046 |

*^1^ Where September = 1, October = 2 etc to reflect the month of entry in UK schooling.*

### Table S3: Associations between teacher expectation and pupil’s polygenic scores (PGS) in complete case sample. Positive values reflect pupils who overperformed relative to their teacher’s expectations, while negative values reflect pupils who underperformed relative to their teacher’s expectations. n=2,239 (KS2); 3,042 (KS3).

|  | Teacher expectation accuracy at Key Stage 2 (age 11) | | Teacher expectation accuracy at Key Stage 3 (age 14) | |
| --- | --- | --- | --- | --- |
|  | Coefficient (95% CI) | P value | Coefficient (95% CI) | P value |
| **PGS** | 0.09 (0.05, 0.12) | <0.001 | 0.07 (0.037, 0.11) | <0.001 |
| **Gender** |  |  |  |  |
| *Female* | *Reference* |  | *Reference* |  |
| *Male* | 0.009 (-0.05, 0.07) | 0.756 | 0.037 (-0.032, 0.10) | 0.293 |
| **Month of delivery^1^** | -0.012 (-0.020, -0.004) | 0.002 | -0.014 (-0.024, -0.005) | 0.004 |
| **SEN status** |  |  |  |  |
| *Not statemented* | *Reference* |  | *Reference* |  |
| *Has a statement* | -0.009 (-0.28, 0.27) | 0.951 | 0.027 (-0.28, 0.34) | 0.863 |
| **Mothers’ highest education** |  |  |  |  |
| *Degree* | *Reference* |  | *Reference* |  |
| *A level* | -0.10 (-0.20, 0.0030) | 0.057 | -0.05 (-0.17, 0.07) | 0.431 |
| *O level* | -0.10 (-0.20, 0.068) | 0.067 | -0.13 (-0.26, -0.005) | 0.042 |
| *Vocational* | -0.27 (-0.41, -0.14) | <0.001 | -0.14 (-0.31, 0.019) | 0.084 |
| *CSE* | -0.30 (-0.43, -0.17) | <0.001 | -0.23 (-0.38, -0.07) | 0.004 |
| **Parental social class** |  |  |  |  |
| *I* | *Reference* |  | *Reference* |  |
| *II* | -0.10 (-0.20, -0.0034) | 0.042 | 0.09 (-0.031, 0.20) | 0.149 |
| *III non-manual* | -0.14 (-0.25, -0.030) | 0.013 | 0.10 (-0.034, 0.23) | 0.145 |
| *III manual* | -0.22 (-0.35, -0.09) | 0.001 | 0.023 (-0.13, 0.18) | 0.774 |
| *IV* | -0.36 (-0.54, -0.18) | <0.001 | -0.007 (-0.22, 0.21) | 0.948 |
| *V* | -0.21 (-0.63, 0.20) | 0.319 | 0.017 (-0.52, 0.55) | 0.952 |
| **Income, £ per week** |  |  |  |  |
| *Over 400* | *Reference* |  | *Reference* |  |
| *300-399* | -0.023 (-0.11, 0.06) | 0.591 | 0.04 (-0.06, 0.14) | 0.395 |
| *200-299* | -0.035 (-0.12, 0.05) | 0.415 | -0.028 (-0.13, 0.07) | 0.577 |
| *100-199* | -0.08 (-0.18, 0.027) | 0.147 | -0.10 (-0.22, 0.018) | 0.094 |
| *Less than 100* | -0.008 (-0.16, 0.14) | 0.911 | -0.17 (-0.35, 0.0012) | 0.052 |
| **Constant** | 0.37 (0.26, 0.49) | <0.001 | 0.18 (0.030, 0.32) | 0.018 |

*^1^ Where September = 1, October = 2 etc to reflect the month of entry in UK schooling.*

### Table S4: Conversion between national curriculum level, point score and fine point score.

| **Sub‐level** | **Point Score** | **Fine point score range, ≤ x <** | **Age at which the UK Government recommend the child achieves this level (years)** |
| --- | --- | --- | --- |
| w | 3 | 0 – 6.4 |  |
| 1c | 7 | 6.4 - 8 |  |
| 1b | 9 | 8 - 10 | 6 |
| 1a | 11 | 10 - 12 |  |
| 2c | 13 | 12 - 14 | 7 |
| 2b | 15 | 14 - 16 | 7 |
| 2a | 17 | 16 - 18 | 7-8 |
| 3c | 19 | 18 - 20 | 7-8 |
| 3b | 21 | 20 - 22 | 7-9 |
| 3a | 23 | 22 - 24 | 7-9 |
| 4c | 25 | 24 - 26 | 9-10 |
| 4b | 27 | 26 - 28 | 11 |
| 4a | 29 | 28 - 30 | 11 |
| 5c | 31 | 30 - 32 | 14 |
| 5b | 33 | 32 - 34 | 14 |
| 5a | 35 | 34 - 36 | 14 |
| 6c | 37 | 36 - 38 | 14 |
| 6b | 39 | 38 - 40 | 14 |
| 6a | 41 | 40 - 42 | 14 |
| 7c | 43 | 42 - 44 |  |
| 7b | 45 | 44 -46 |  |
| 7a | 47 | 46 - 48 |  |
| 8c | 49 | 48 - 50 |  |

Details on conversion from level to scores available at https://dera.ioe.ac.uk/26474/1/Key_stage_1_and_2_test_and_examination_point_scores.pdf

### Figure S1: Flowchart detailing inclusion and exclusion criteria


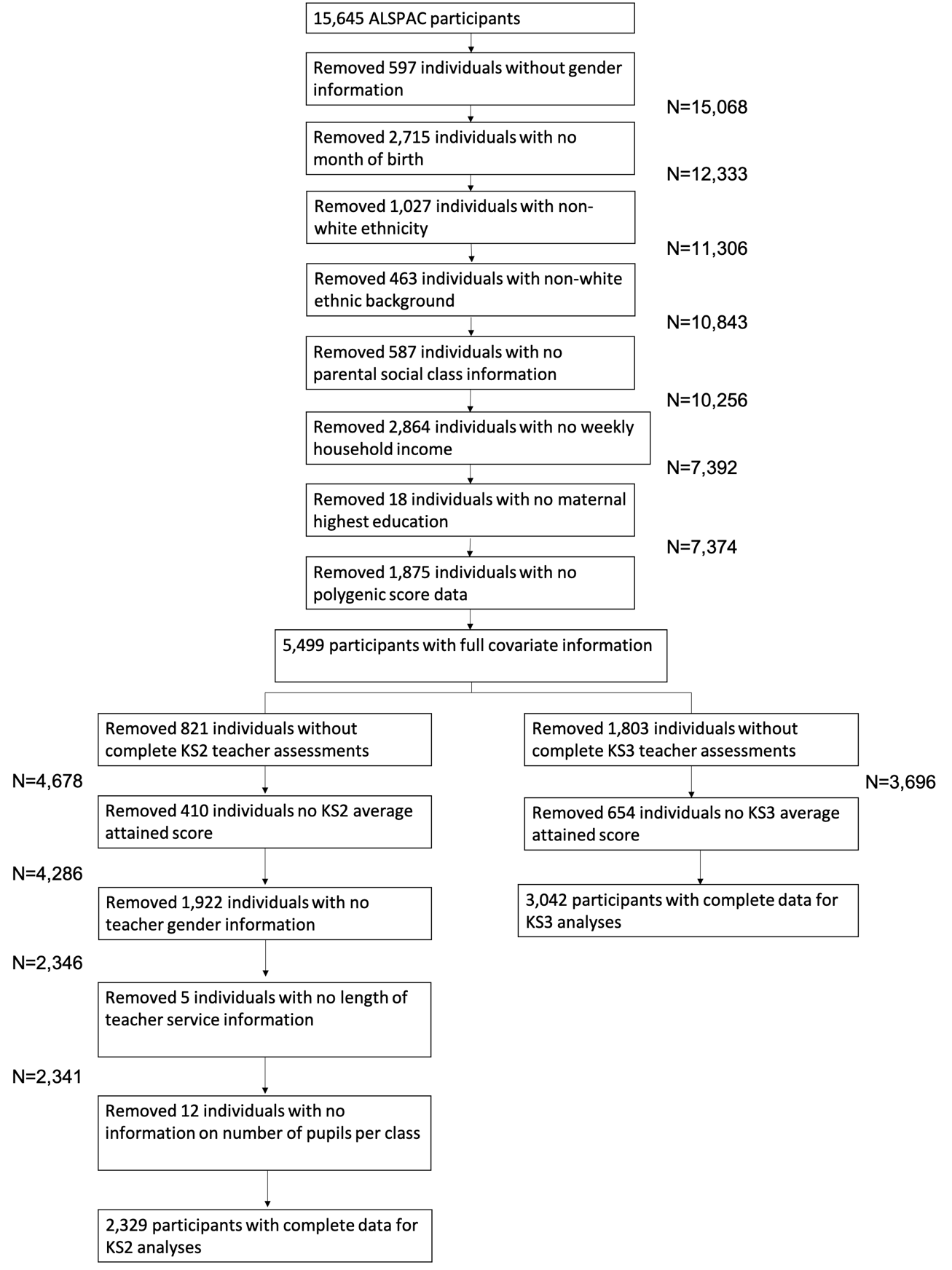

Supplement: Supplementary file 1 — Supplementary Information. [file 41598_2022_11347_MOESM1_ESM.docx]
